# Supplementary figures and images for: Retrieval of contextual memory can be predicted by CA3 remapping and is differentially influenced by NMDAR activity in rat hippocampus subregions
Source: PLoS Biol. 2024 Jul 1;22(7):e3002706. doi: 10.1371/journal.pbio.3002706 (PMC11244845; doi:10.1371/journal.pbio.3002706)

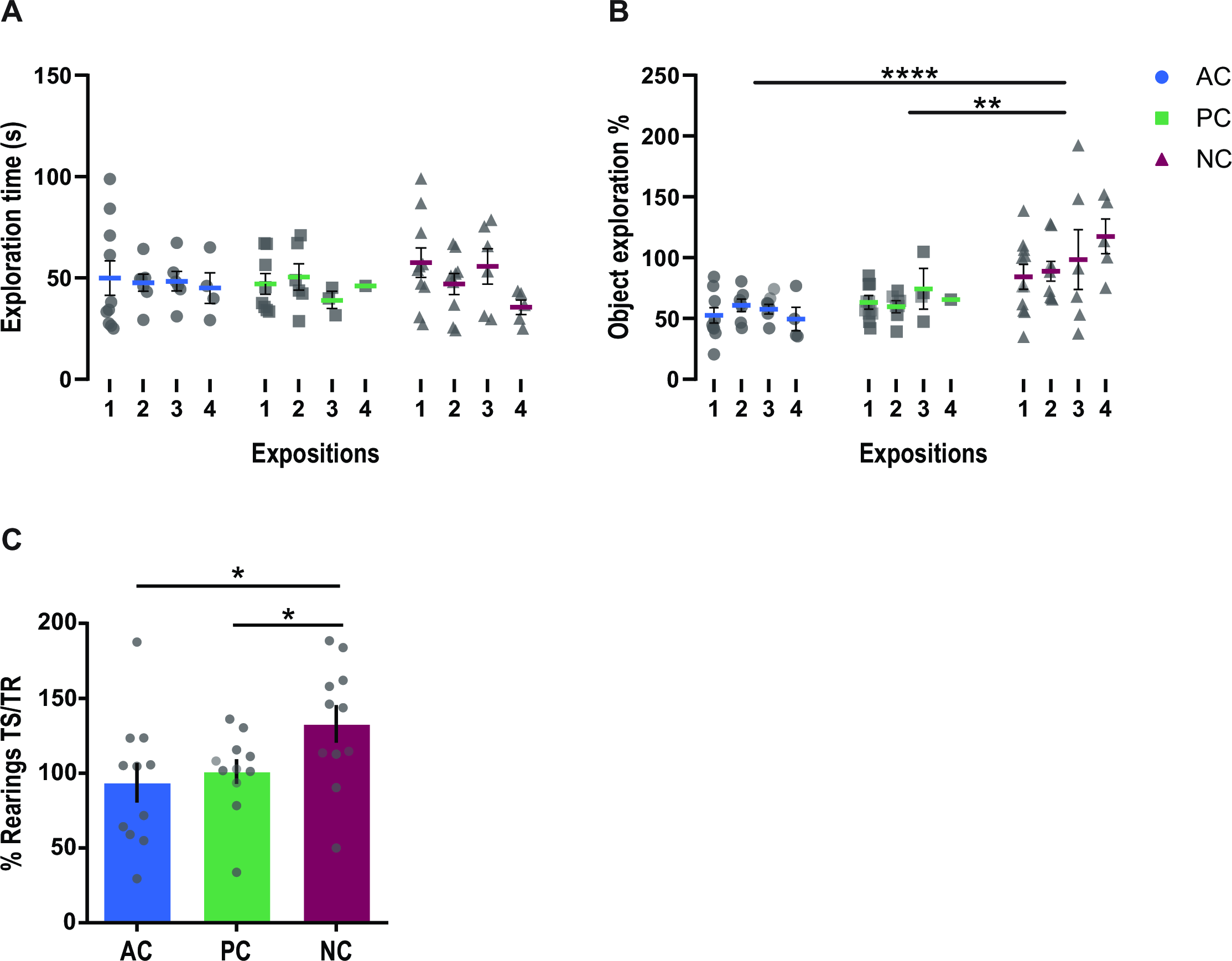

Supplement: S1 Fig — (A) and (B) Repeated measure analysis. (A) Total object exploration during the training session for AC, PC, and NC in the different expositions to the same condition during the 1-day version of the task. Two-way RM ANOVA, n = 10, sessions = 75, each dot represents a session, interaction: F = 0.5755, p = 0.7484. AC: 47.89 ± 3.38, PC: 47.39 ± 3.07, NC: 49.74 ± 3.50. (B) Total object exploration percentage for AC, PC, and NC in the different expositions to the same condition during the 1-day version of the task. Two-way RM ANOVA, n = 10, sessions = 75, each dot represents a session, F = 16.04, p < 0.0001.Tukey’s post hoc test: **** p < 0.0001 AC vs. NC; p = 0.25 AC vs. PC; ** p = 0.001 NC vs. PC. One sample t test against 100% AC t = 14.45, p < 0.0001; PC t = 8.76, p < 0.0001; NC t = 0.37, p = 0.91. AC: 56.98 ± 3.31, PC: 63.93 ± 4.11, NC: 92.13 ± 7.34. The re-exposures to the different conditions have no effect on the exploration time in the training session or in the object exploration percentage. (C) Percentage of rearings during the test phase in relation to training for the AC, PC, and NC conditions in the 2-day version. One-way RM ANOVA F = 4.26, p = 0.048, AC: 93.57 ± 13.18, PC: 101.10 ± 8.28, SC: 132.9 ± 12.53. n = 11. * p < 0.1. While no previous differences between groups in rearings were present during the training session (RM one-way ANOVA, F = 0.30, p = 0.69), post hoc comparisons revealed a marginally significant increase in the NC condition when compared to both the AC and PC conditions (AC vs. NC: t = 2.23, p = 0.05, NC vs. PC: t = 2.14, p = 0.06). To note, unlike object-related exploration, contextual exploration in the AC condition (as manifested by the number of rearings) does not decrease between training and test since animals were habituated to the AC context (and not to the object) prior to training session (one sample t test from 100%, AC t = 0.49, p = 0.636, PC t = 0.13, p = 0.896, NC t = 2.62, p = 0.025). Individual values used to calculate the [file pbio.3002706.s001.tif]

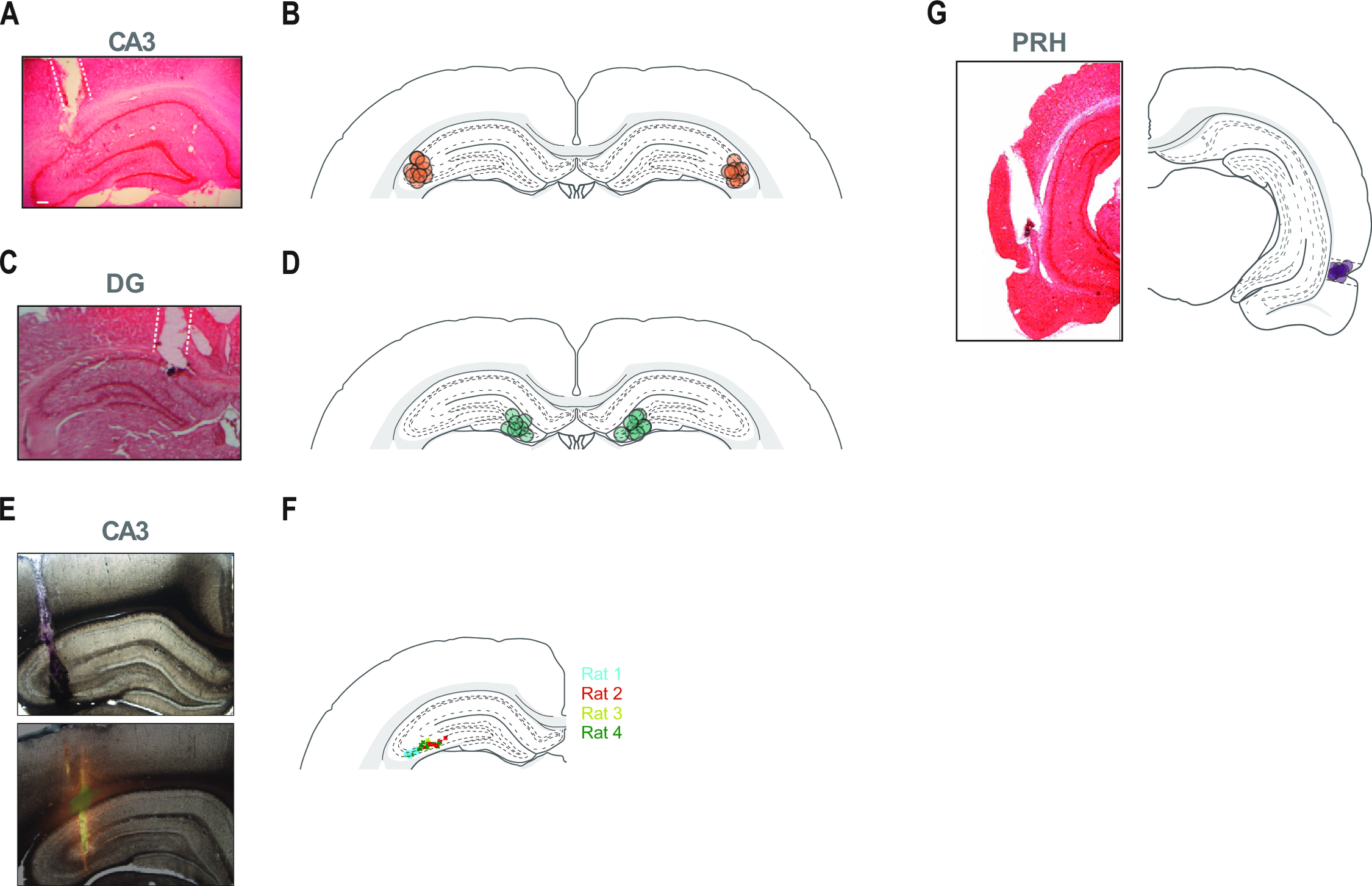

Supplement: S2 Fig — (A) Safranin staining image of a coronal brain section of a rat cannulated in the CA3 of HP. (B) Schematic representation of coronal sections of the rat brain with representative infusion area in an example experiment. (C) Safranin staining image of a coronal brain section of a rat cannulated in the CA3 of HP. (D) Schematic representation of coronal sections of the rat brain with representative infusion area in an example experiment. (E) Two representative coronal brain sections with tetrode trajectory. (F) Schematic representation of the tetrode recording sites for 4 recorded rats in CA3. Each cross represents a tetrode tip. See full methods for verification of tetrode recording sites. (G) Safranin staining image of a coronal brain section of a rat cannulated in the PRH. Scale: 200 μm and schematic representation of coronal sections of the rat brain with representative infusion area in an example experiment. All schematic representations of brain slices are adapted from Swanson’s Brain map: structure of the rat brain (Swanson, 2004), levels 32 and 37, which is distributed under the terms of a Creative Commons Attribution-Noncommercial 4.0 license CC BY-NC-4.0 (https://creativecommons.org/licenses/by-nc/4.0/). As such it is not covered by the CC BY 4.0 license and further reproduction of these panels would need to follow the terms of the CC BY-NC 4.0 license. The data that support these findings is available in OSF at https://osf.io/7pw23/. (TIF) [file pbio.3002706.s002.tif]

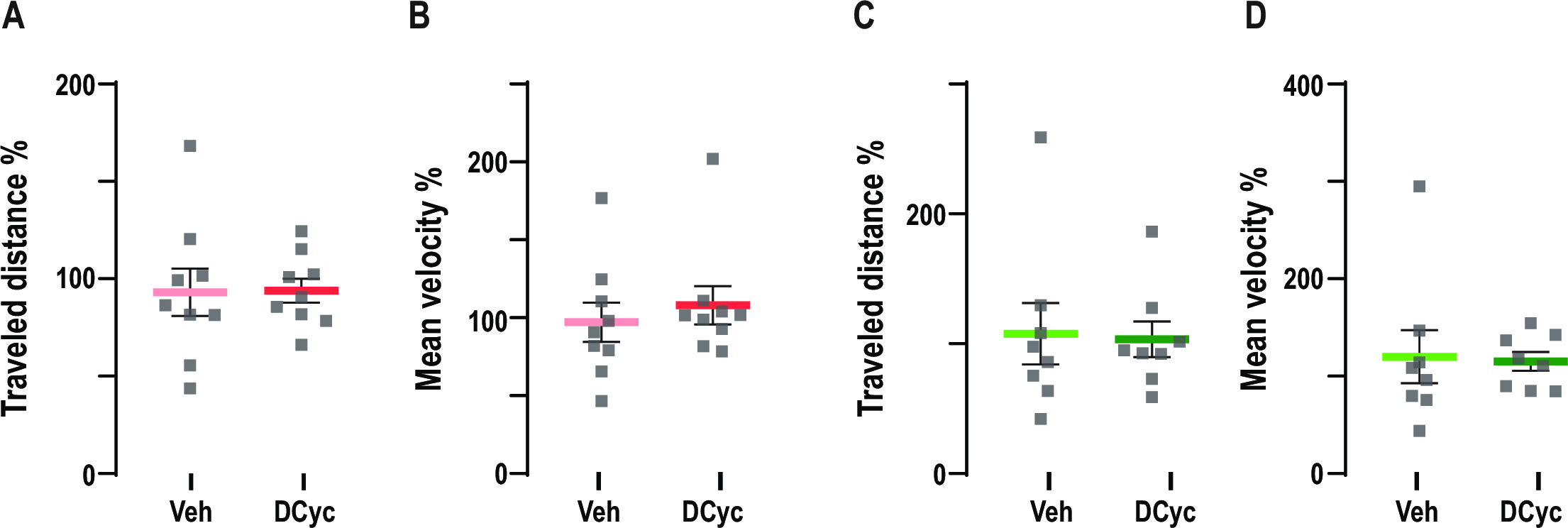

Supplement: S3 Fig — (A) Percentage of total distance traveled during test on Fig 5F. No significant difference was found in the distance traveled by animals after infusion of DCyc in CA3 compared to Veh. Paired t test, p = 0.949, t = 0.06. Veh: 93.11 ± 12.16, DCyc: 93.90 ± 6.18. (B) Percentage of average speed in cm per second of animals injected in CA3 with DCyc compared with Veh. Paired t test, p = 0.548, t = 0.63. Veh: 97.10 ± 12.60, DCyc: 108.00 ± 12.26. (C) Percentage of total distance traveled during test over training in the experiment in Fig 5E. No significant differences were found between the distance traveled by animals after infusion of DCyc in the DG compared to Veh. Paired t test, p = 0.877, t = 0.16. Veh: 107.7 ± 23.60, DCyc: 103.3 ± 13.81. (D) Percentage of average velocity in cm per second during the test over training in Fig 5E. No significant difference was found in the speed of animals injected in the DG with DCyc compared to Veh. Paired t test, p = 0.841, t = 0.21. Veh: 120.00 ± 27.17, DCyc:115.2 ± 9.71. The data that support these findings is available in OSF at https://osf.io/7pw23/. (TIF) [file pbio.3002706.s003.tif]

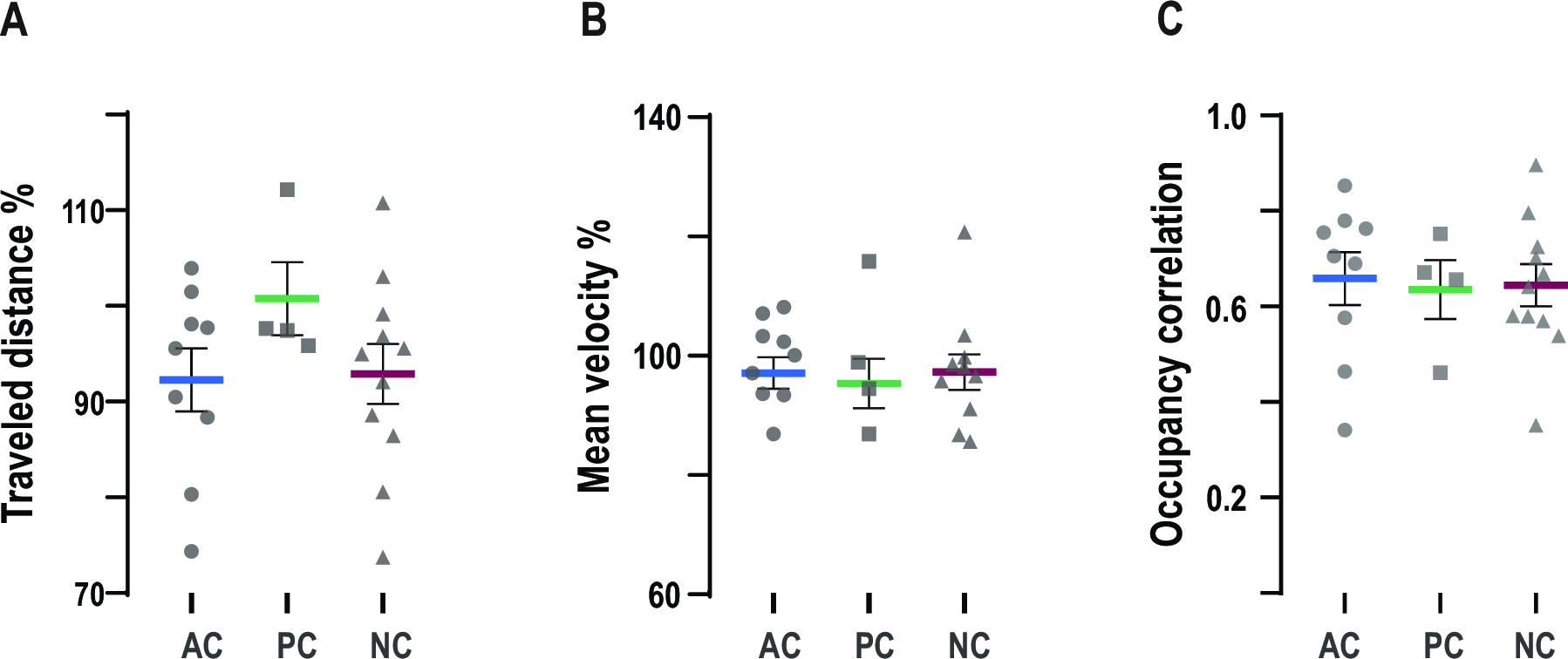

Supplement: S4 Fig — (A) Percentage of total distance traveled during test over training in the experiment in Fig 3. No significant differences were found between the distance traveled in each condition, n = 24, one-way ANOVA, p = 0.62. Tukey’s post hoc test: p = 0.99 AC vs. NC; p = 0.33 AC vs. PC; p = 0.37 NC vs. PC. AC: 92.26 ± 3.29, PC: 100.8 ± 3.81, NC: 92.89 ± 3.11. (B) Percentage of average velocity in cm per second during the test over training in Fig 3. No significant difference was found in the speed of animals between conditions, n = 24, one-way ANOVA, p = 0.93. Tukey’s post hoc test: p = 0.98 AC vs. NC; p = 0.93 AC vs. PC; p = 0.97 NC vs. PC. AC: 93.91 ± 0.03, PC: 92.17 ± 0.04, NC: 94.07 ± 0.03. (C) Pearson correlation between the occupancy maps of the training and test phase. No significant difference was found in the occupancy maps of the training and test phases between conditions, n = 24, one-way ANOVA, p = 0.91. Tukey’s post hoc test: p = 0.94 AC vs. NC; p = 0.99 AC vs. PC; p = 0.93 NC vs. PC. AC: 0.66 ± 0.06, PC: 0.64 ± 0.06, NC: 0.64 ± 0.04. The data that support these findings is available in OSF at https://osf.io/7pw23/. (TIF) [file pbio.3002706.s004.tif]

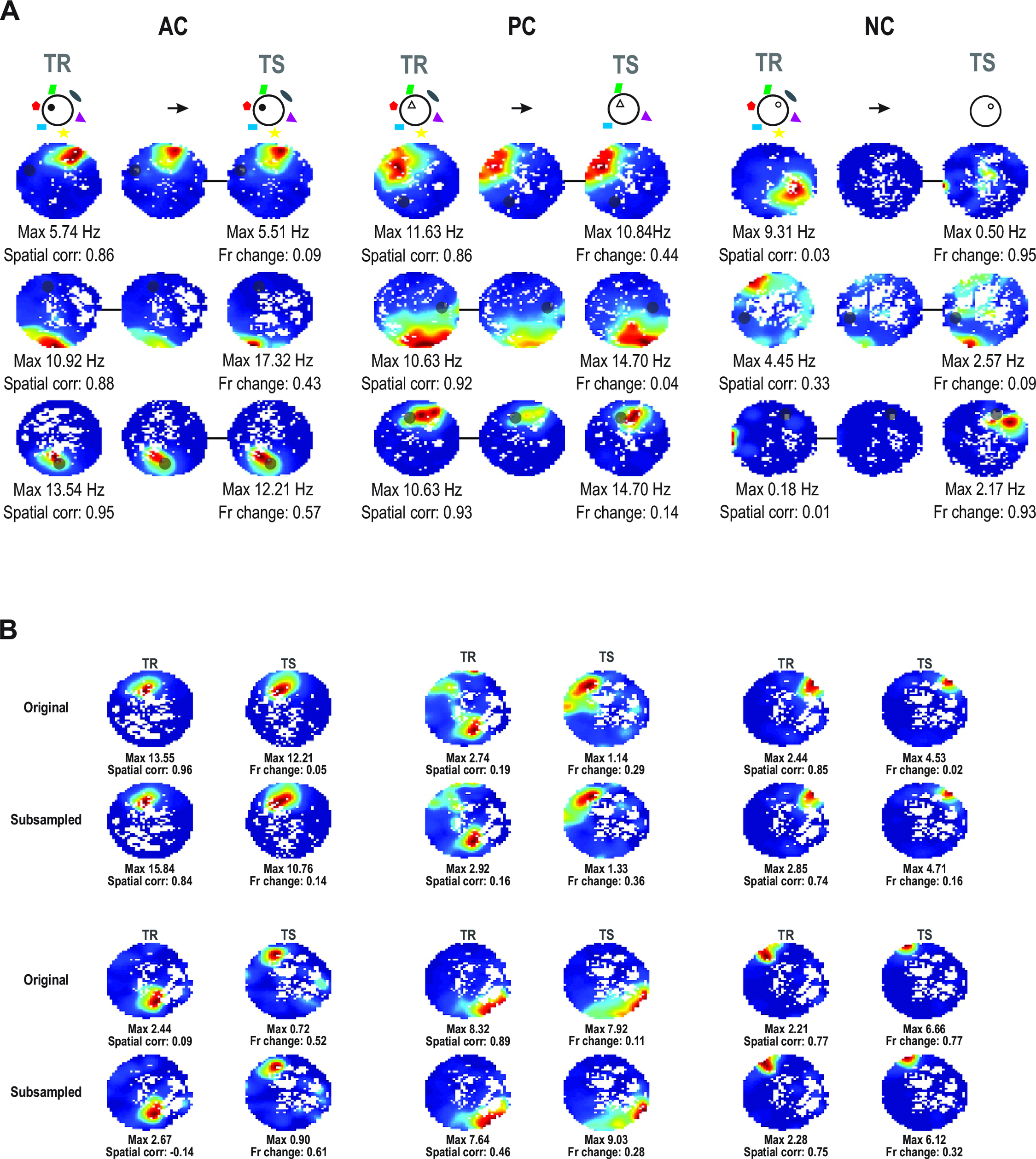

Supplement: S5 Fig — (A) Normalized firing maps of Fig 3. Place maps for training session (left) and test session (right) for each condition. Extreme’s firing maps were normalized by the max firing rate of each neuron and the middle firing map was normalized by the max firing rate of the phase with higher firing rate peak. Spatial correlation and firing rate change were calculated for each neuron. (B) Original and subsampled firing maps. Subsampled firing maps were constructed by down-sampling the spike and position data in each spatial bin to match the minimum number of samples in the less visited bin of the training or test. The data that support these findings is available in OSF at https://osf.io/7pw23/. (TIF) [file pbio.3002706.s005.tif]

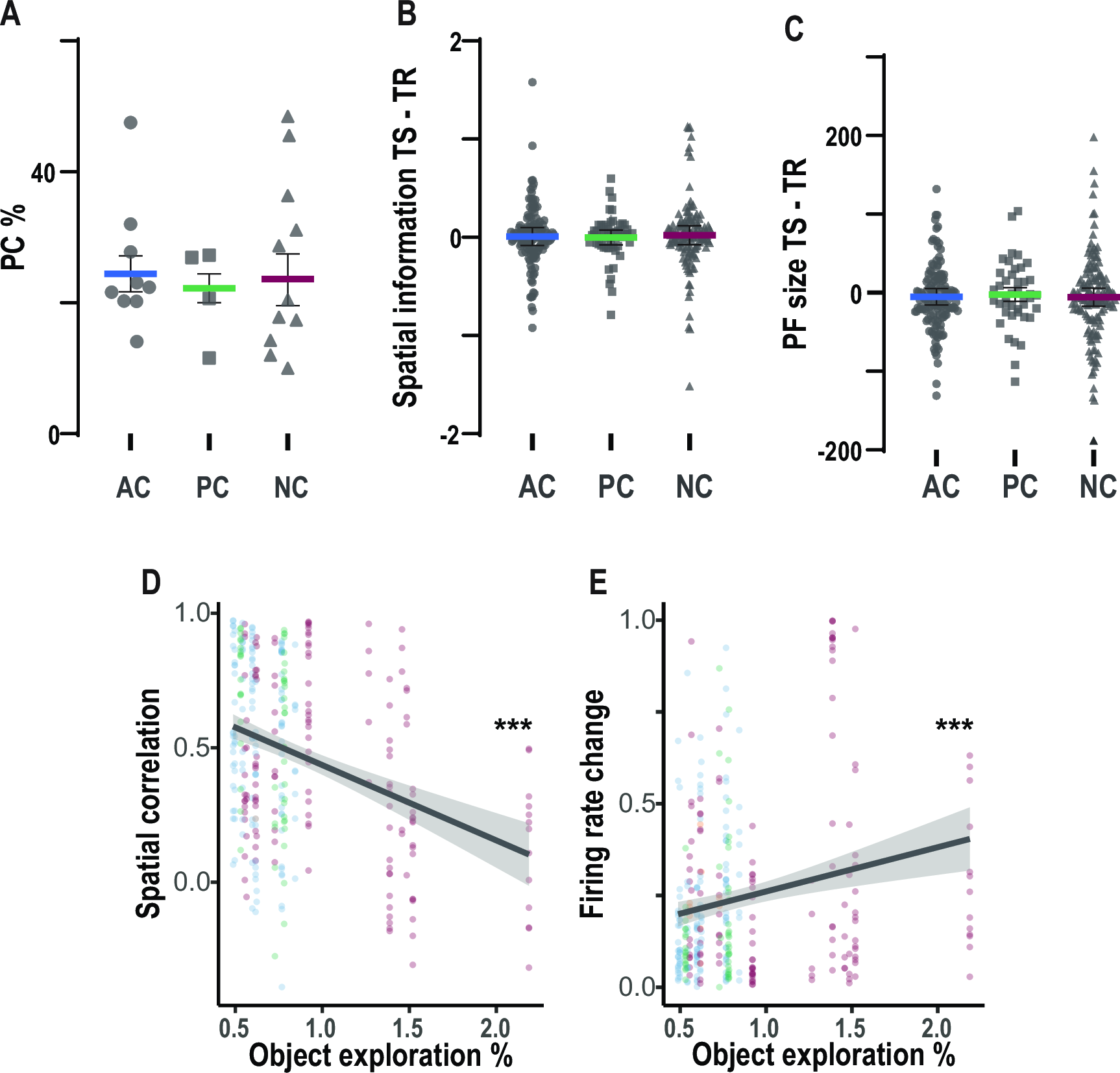

Supplement: S6 Fig — (A) Percentage of place cells over the total number of recorded neurons in each session. No significant differences were found between conditions. n = 24, Kruskal–Wallis test p = 0.99 AC vs. NC; p = 0.86, AC vs. PC; p = 0.88 NC vs. PC. AC: 25.65 ± 3.21, PC: 21.59 ± 3.84, NC: 25.22 ± 4.86. Context enrichment does not increase the proportions of place cells coding a context. (B) Difference in spatial information index between test and training. No change in spatial coding between conditions. n = 54–141, Kruskal–Wallis test NC-AC p = 0.93, NC-PC p = 0.88, and AC-PC p = 0.98. AC: 0.01 ± 0.03, PC: 0 ± 0.03, NC: 0.02 ± 0.03. (C) Difference in place field size between test and training. No significant differences in place field size between conditions. n = 54–141, Kruskal–Wallis test NC-AC p = 0.96, NC-PC p = 0.78, and AC-PC p = 0.67. AC: −5.21 ± 3.43, PC: −2.43 ± 6.58, NC: −5.59 ± 4.37. (D) and (E) Correlation between place cell activity and memory output. Spatial correlation: Pearson’s correlation between spatial correlation and object exploration %, n = 332, p = 1.47e-10, R = −0.34. Firing rate change: Pearson’s correlation between firing rate change and object exploration %, n = 332, p = 0.00015, R = 0.20. The data that support these findings is available in OSF at https://osf.io/7pw23/. (TIF) [file pbio.3002706.s006.tif]

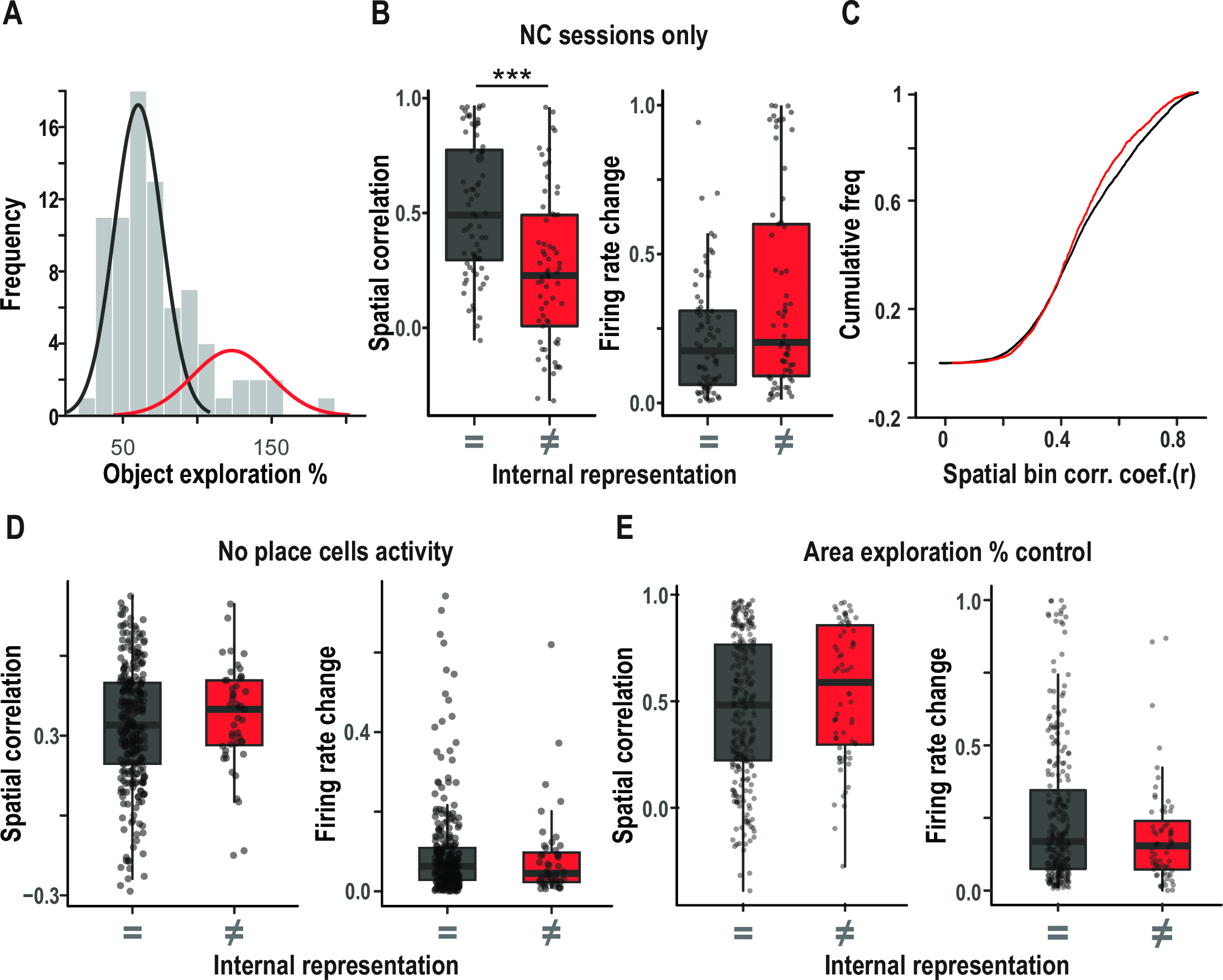

Supplement: S7 Fig — (A) Object exploration percentage histogram. Gray line represents the normal distribution of the = sessions, red line represents the normal distribution of the ≠ sessions. (B) Spatial correlation (left) and firing rate change (right) sorted by internal representation using only place cells activity from NC condition, n = 65–72, LMM, p spatial correlation = 0.0007, =: 0.52 ± 0.03, ≠:0.25 ± 0.04; p firing rate change = 0.1742, =: 0.22 ± 0.02, ≠:0.36 ± 0.04. (C) Population vector. The population vector analysis follows the same tendency that the single unit analysis. (D) Non-place cells have not a role encoding animal internal representation of context. A number of neurons equal to the one in Fig 3 was selected randomly from the subset of recorded neurons that did not fulfill place cell criteria and an analysis equivalent to the one in Fig 3 was applied. Right: No place cell spatial correlation sorted by internal representation. n = 65–267, LMM, p = 0.48, =: 0.34 ± 0.01, ≠:0.37 ± 0.03; left: no place cell firing rate change sorted by internal representation. n = 65–267, LMM, p = 0.37, =: 0.10 ± 0.01, ≠:0.08 ± 0.02. (E) Place cells activity sorted by a threshold determined by the exploration of an area opposite the object. Right: spatial correlation sorted by control internal representation. n = 65–267, LMM, p = 0.87, =: 0.46 ± 0.02, ≠:0.54 ± 0.03. Left: firing rate change sorted by control internal representation. n = 65–267, LMM, p = 0.78, =: 0.26 ± 0.02, ≠:0.19 ± 0.02. The data that support these findings is available in OSF at https://osf.io/7pw23/. (TIF) [file pbio.3002706.s007.tif]
