# Supplementary material for: Retrieval of contextual memory can be predicted by CA3 remapping and is differentially influenced by NMDAR activity in rat hippocampus subregions
Source: PLoS Biol. 2024 Jul 1;22(7):e3002706. doi: 10.1371/journal.pbio.3002706 (PMC11244845; doi:10.1371/journal.pbio.3002706)
Supplement: Attachment — Submitted filename: FINAL Response Reviewers Plos Biology with Citations.docx [file pbio.3002706.s008.docx]

Manuscript number: PBIOLOGY-D-23-02575R1

Article title: Behavioral, molecular and neuronal mechanisms involved in spatial recognition memory retrieval in the rat hippocampus

RESPONSE TO REVIEWERS

The following response has been prepared to address the Reviewers comments in a point-to-point fashion. All modifications introduced in our revised manuscript are highlighted in yellow below and all new references have been duly incorporated into the “Bibliography” section.

**RESPONSE TO REVIEWER 1**

**Comment 1.1:** Understanding whether and how contextual representations guide memory retrieval is a fundamental question in neuroscience which must be addressed at multiple levels of explanation - from synapses to circuits. Here, Miranda et al. add to our knowledge of this process by presenting various pieces of causal and correlational evidence linking protein synthesis, NMDA receptor activation, single-unit activity, and behavioral assays of context-object memory in rats.

The workhorse of this study is a paired object-context learning paradigm, with object exploration times serving as the dependent of object memory, and manipulations to the cues which comprise the context. When exposed to just a subset of visual cues previously present during a context-object association (partial cue - PC), behavioral evidence indicates that an internal representation of context is retrieved despite degraded sensory cues. Interfering with protein synthesis in perirhinal cortex following a second exposure to the context of a context-object association disrupts the memory for the paired object, but does not disrupt the memory for objects encountered in other environments. Electrophysiological recording of place cells in CA3 indicates that the hippocampal map remains stable even in the degraded PC condition, and that the amount of remapping in CA3 in an extra-degraded no-cues condition correlates with the behavioral evidence of a change in the internal representation of context.

**Response 1.1:** We thank the reviewer for his/her careful reading of our work. We are glad that the reviewer found our narrative clear, our study exhaustive and our conclusions sensible.

**Comment 1.2:** For the CA3 electrophysiology results, I would consider including analysis of population vector similarity to complement your findings. A population vector measure summarizes the similarity of the neural population as a whole recorded in a session, and would allow you to perform an animal-level analysis rather than treating each place cell as independent. This might clarify some of the variability you observe across cells.

**Response 1.2:** We appreciate the Reviewer’s comment, which has allowed us to better define this important aspect of our study. We have included the population vector analysis in Fig. S7. As we found in the single-unit analysis, the population vectors corresponding to sessions in which animals discriminated between contexts (different internal representation) were less similar than the population vector of sessions with similar internal representation. We also commented this result in the results section as following:

*“The differences in the spatial coding between phases of the task was also analyzed using a population vector analysis (Fig S.7C). This analysis showed the same tendency as the one observed at the level of single units, where the spatial coding is more stable in non-retrieval sessions.”* (see page 5, line 265)

The effects on the spatial correlations or the firing rate chance reported were compared using a linear model that included the animal as a random effect, and we found that the animal identity does not significantly explain the variation in the response. This was clarified in the methods section.

Related to this comment, we have included a new analysis, as proposed by Reviewer 3, where we classified cells based on their spatial correlation and firing rate change (Figure 3H-J). Two clusters were identified. One cluster has neurons with low spatial correlation and high firing rate change. In most sessions where animals had a different internal representation of the context, neurons from this cluster represented the vast majority of cells. Conversely, the other cluster consisted of neurons with high spatial correlation and low firing rate changes, and they constituted the majority of the data in sessions where animals were able to retrieve.

**Comment 1.3:** For your internal representation GLM - the model with more variables fits best, but perhaps needs to be controlled for the number of variables (2 > 1). Maybe compared to a shuffled version of the variable of interest, and/or report the marginal gain in R2?

**Response 1.3:** We welcome the Reviewer’s insightful suggestion. Indeed, we believe that incorporating metrics that better control for the number of variables is important for this analysis. We have now added the Akaike information criteria (AIC) as metrics for comparing between the models. It is important to note that, in addition to the residual deviance, the AIC score also includes the number of variables as a parameter in its formula. In other words, it penalizes models that require many parameters to achieve a good fit to the data, favoring simpler models. Also, we clarified the anova analysis that we did to compare models including 1 variable versus models including 2 variables. Both analyses suggest that the model that includes spatial correlation and firing rate change is the best to explain our data. We commented this result in the results section as following:

*“The best of the three models is the one that includes both variables (AIC 1-variable model= 294.30 spatial corr, 314.01 fr change, AIC 2-variables model = 291.16; and ANOVA comparing models with increasing variables, p 1-variable model vs p 2-variables model = 0.009).”* (see page 6, line 272)

**Comment 1.4:** For the Figure 4 caption and related text, the acronyms TC and CP are used. I'm not sure if I'm missing their reference or if they are referring to AC and PC conditions, respectively (on my reading, I assumed the latter). This might be me making a mistake but I would double-check those referents and/or clarify their meaning as they occur.

**Response 1.4:** We thank the Reviewer for pointing out this mistake. Indeed, the correct acronyms are AC and PC. We have thoroughly searched for mistakes in the acronyms throughout the manuscript to AC and PC and corrected them.

**RESPONSE TO REVIEWER 2**

**Comment 2.1:**  In this study, the authors exposed rodents to differently enriched environments and tested their exploration time of a familiar object in relation to a novel object, which animals have never seen before. They find that in dependence on the number of external cues surrounding the environment the recall of a context and the differentiation between the familiar and novel object is higher in enriched contexts (ACs) than in poor in 'non cued' (NC) environments.

Response 2.1: We thank the reviewer for thoroughly reading our MS and providing insightful comments to improve our work. However, we found a few misconceptions in the reviewer’s interpretation of the data and the results, perhaps because it requires further clarification. Some of the reviewer descriptions of the study do not match the results or the conclusions of the experiments presented in the study. For example, the reviewer indicates that “*the authors exposed rodents to differently enriched environments and tested their exploration time of a familiar object in relation to a novel object, which animals have never seen before*”. We want to clarify that, while one might consider that the contexts with 6 visual cues are more “enriched” than the ones with half the cues, we chose not to use these wording which can be misleading. Enriched environments usually include different types of stimulation, such as physical, olfactory or tactile. In the present study, the only change between one context and the other is the number of visual cues, all the rest of the cues remain unchanged between successive exposures. Also, in most of the experiments, we do not compare exploration times between a familiar object and a novel object, but between the same object in contexts that vary in the surrounding (removable) visual cues (see experiments depicted in Figures 1, 3, 4 A-C, 5). Only in the reconsolidation experiments in which we inject Emetine in the perirhinal cortex, we eventually compare exploration times between familiar and novel objects, but in a context different from the ones used in the spatial part of the task (see Figures 2, 4D). For a more detailed explanation as to why the experiments were designed in this way, see response to comment 2.3.

**Comment 2.2:** Next, they block protein synthesis by injecting Emetine in the perirhinal cortex and find that the discrimination between the familiar and novel object is only reduced in the cue-enriched AC context but not the non-cued NC context. The authors conclude first, that protein synthesis during consolidation is needed for the recall of object-context memory; and (2) that contexts are capable of guiding reactivation of 'relevant' object memory, while memories of 'non-relevant objects' are not susceptible to the action of protein synthesis inhibitors (page 4, second paragraph). This conclusion is an over-interpretation of the data and actually not supported by the data. What is the definition on a 'relevant' and 'not-relevant memory'? What seem to matter is the order of context presentations. If a cue-enriched context is presented a second time after the initial object-context pairing than the animal memorizes in the test phase the familiar object and explores the novel one. The second exposure to the enriched context seems to be important to support the association in the object-context memory. If the animal is exposed to an empty arena (NC context) than the association between object and the initial context seemed to be markedly reduced. However, this has nothing to do with relevant or on-relevant object memory

Response 2.2. The reviewer indicates that *“(1) The authors conclude first, that protein synthesis during consolidation is needed for the recall of object-context memory*”. This is not the conclusion of that experiment at all. First, we did not intend to block memory **consolidation** but rather memory **reconsolidation**. This is achieved by injecting the protein synthesis inhibitor right **after** the long-term memory test session. This manipulation does not affect the original consolidation of the object-in-context memory. Regarding conclusion number (2), we believe we might be describing the same phenomenon but using different wording. To us, the fact that the animal recalls an association between a given context and an object indicates that the association was **relevant** to that particular event (ie. context only exposure), but not to other events that do not include elements of this specific association. However, we understand this wording could lead to misunderstanding the meaning of the results and thus, we have changed the wording to eliminate any judgment about the relevancy of the object memory trace, as following:

*“Additionally, this suggests that all the contexts by themselves are capable of guiding reactivation of an object memory originally associated with that context, leaving the memories of objects linked to a different context unaffected by the action of protein synthesis inhibitors.”* (see page 4, line 185)

There is no way with these data to define whether the association between object and context is reinforced or reduced during TS1 because we do not behaviorally test the association between object and context at this stage (e.g. with a third exposure to the object in one of the contexts). Indeed, on TS2, we only test the object memory, by placing the two different familiar objects against a novel one in a different context. Therefore, changes in the association between the object and the context cannot explain our results. During this TS2, we found no difference in Vehicle injected animals in terms of discrimination ratio of the two familiar objects between groups that could indicate that context exposure in the absence of any object affects by itself the object memory.

We believe it’s important to emphasize that in most experiments, like the one depicted in Figure 1, the animals experienced the AC context containing an object in the TR. In the test phase (TS), the animals experience one of the possible contexts (AC, PC or NC) that contain the **same object** seen during TR. There is no discrimination index between novel and familiar object, since the object does not vary between TR and TS. The difference in exploration time is driven by the combination of the object and the context. If the animal perceives this combination as novel, it will explore the object roughly the same time during TR and TS. On the contrary, if it is perceived as familiar, the animal will explore less during TS than during TR.

**Comment 2.3:** Why was protein synthesis blocked in the perirhinal cortex and not e.g. in the dentate gyrus well known to store information for a live time (see below)?

Response 2.3: Perhaps we were not clear enough when we explained the aim of these experiments. The perirhinal cortex is a canonical structure in the medial temporal lobe for information storage regarding object identity [1, 2]. The role of the dentate gyrus in object information storage has not been thoroughly clarified [3], but its role in differentiation of spatial memories is more established [4-9]. Based on this information, we wanted to know whether a second exposure to the context only, could lead to reactivation of a particular object-context experience. The reactivation of this experience includes that of the identity of the object. If the context alone was sufficient to reactivate the whole object-context experience, then we predicted that the representation of the object would be reactivated in the perirhinal cortex and thus would be susceptible to disruption by a protein synthesis inhibitor in this particular region. Any manipulation of the hippocampus could have affected the reactivation of the contextual part of the representation, and any result would have not responded to our original inquiry.

We might have not been clear enough about the conditions of our reconsolidation experiments. For example, in the protocol used for experiments in Figure 2 C, for both the AC and NC group, the original object in context experience is exactly the same (day 1, TR on cued spatial context) and so is the second test session on day 3 on a triangular context where there are no spatial cues (TS2). Moreover, the two contexts used during day 1 and the two tests performed on the triangle on day 3 are presented in a counterbalanced fashion. Therefore, the order of contextual exposure cannot explain the results found. The only difference between groups is on day 2 during TS1. At this point, the AC group will re-experience the empty context in its original form, while the NC group will re-experience it in the absence of contextual cues.

Critically, during this re-exposure, **there is no object present**. Therefore, the animal cannot ‘memorize’ during the test phase the familiar object unless the original object in context memory is reactivated. When the animals re-experience the context without any object on TS1, this contextual experience could or could not lead to the reactivation of their prior experience (TR) where this context (and either the A or C object) were also present.

In the absence of Emetine, a re-experiencing of the context (independently of whether it is an AC or NC context) has no effect over the object memories on TS2 (see Fig.2E, white columns). Indeed, the object discrimination ratio of both familiar object A against novel object B (1st and 5th column), and of familiar object C against novel object D (3rd and 7th column) is not different between animals that experienced the empty context AC or the empty context NC in the absence of Emetine. This would be expected since in both cases the object is familiar (shown during training) and the two groups of animals only differ on their contextual experience on TS1, but not on their object experience. It is important to clarify that during the TS2 we are not evaluating a context-object association, but only the memory of the identity of objects A and C. This is because the objects are presented in a novel triangular context where they were never experienced before. This is done with the intention to have a measure of the object memory independently of the contexts in which it was experienced before.

If the exposure to an empty arena by itself affected the memory of the object, as the reviewer suggested, this should translate into a change in the object discrimination index in animals injected with Veh. However, we do not see any evidence of this. On the contrary, what we do find is that only the presence of Emetine during the re-exposure to an AC context -without any object present- affects the memory of an object that was experienced one day before in association with the AC context.

The original training experience on day 1 is the only moment where the animals were in contact with the object. We deduced that the context is guiding the reactivation of object memories, because only the context is serving as a cue for retrieval/reactivation during TS1. Because there is no object during TS1 exposure, only if the context itself can guide the retrieval of the object memory, this object memory will be susceptible to protein synthesis inhibitors administered immediately after TS1. Indeed, previous work from our team has shown that retrieval of object memories in object in context tasks is context guided [10]. We used the word ‘relevant’ for the object memories that appear to be susceptible to Emetine, which would be the stronger memory for that particular situation or the context appropriate memory (and not the other object memory). In addition, the order in the object test sessions could not have affected our results, since it was counterbalanced for all groups (see Methods). We have added this information in the Results section for better clarity (Page 4, line 174).

We have also added information in the Methods section making emphasis on the absence of any object during the TS1 session, and explaining in more detail the protocol used for Fig. 2 and 4D. Additionally, we have clarified the role of the TS2 test on the triangular object as a way to assess object memory independently of contextual information, as following:

*“For reconsolidation experiments (Fig. 2,4C), a 3-day protocol was used. First, rats were habituated in both the open field for 5 days and in a triangular arena for 3 days. After habituation, on day 1, animals were trained by placing an object in the AC context for 10 min as previously and, 24 hours later, a retrieval session was performed. During this session (TS1) on day 2, animals were exposed to an empty context (without any object present) for 10 min with a variable number of contextual cues (depending on the experiment). During a second test session (TS2) 24 hours after (day 3), a copy of the original object was presented next to a novel object in the triangular arena for 5 min. For the experiment in Fig. 2C, during the first day, animals were trained in the AC context with an object A and in the NC condition with the object B. These two training sessions were given in a pseudorandom order and separated by 2 h. During the evaluation, a group was exposed to an empty AC context with all the cues, and another group was exposed to the empty AC context without cues. In all cases, the empty context corresponds to a context without any object present. In a second evaluation, memory for the presented objects (A or B) was tested against two different novel objects (C or D) in the triangular arena for 5 min. The triangular arena was made of white foam board and lacked any proximal spatial cues. Each wall was 60 cm long by 60 cm high to prevent the use of distal spatial cues. In this way, for this final day, the memory of two familiar objects (experienced originally in different contexts) is tested against two novel ones in a novel triangular context. The order of the two test sessions was counterbalanced.*

*For experiments on Fig 2A and 4D, after training on a context-object association as before (day 1), were subjected to an AC, PC or NC contextual re-exposure in the absence of any object, and Emetine or Veh were infused in the Prh immediately after (TS1). On day 3, the original object memory of day 1 was tested on the triangular context against a novel object (TS2).”* (see page 12, line 641)

**Comment 2.4:** Next, the authors perform single unit recordings within CA3 during exploration of enriched contexts (TR 6 cues vs TR, 6 cues; TR 6 cues versus TR 3 cues; TR 6 cues versus no cue), and find relationships between the three different context comparisons and spatial parameters such as spatial tuning and exploration time of the object. What is missing is the number of place cells and place field correlations.

Response 2.4: We thank the reviewer for pointing this out, we specified the number of place cells in the figure legend.

**Comment 2.5:** Finally, the authors apply AP5 infusion to block NMDA receptors in the dentate gyrus or the CA3 area and show that this interference causes the reduced animals' ability to discriminate between objects (pattern separation) a well-known phenomenon previously observed by McHugh et al (Nature 2007). In summary, most of the findings relate to previously published findings, which lowered my enthusiasm for the study.

Response 2.5: We respectfully disagree with the reviewer regarding the novelty of our work. Our study, indeed, was greatly inspired by the publication mentioned by the reviewer, which, of course, we found important for our own project. We believe that our work is novel and highly relevant since, although some studies have shown how different experiences can be represented independently in the hippocampus [11, 12], there have been almost no attempts to provide a direct link between the presence of external retrieval, the individual’s internal context representation and the behavioral output. As we mention in the manuscript, the role of NMDAR in pattern separation was studied by *McHugh et al. (2007)*, however there has never been an attempt to understand how this role could alter the balance between memory differentiation and discrimination. The use of knock out mice in these seminal studies [5, 13], did not allow the distinction between the effects of NMDARs manipulation on memory retrieval and its effects on memory acquisition, giving no information on their particular role in retrieval of specific experiences or encoding of novel experiences. Here, we show that NMDARs in the DG not only have an impact on memory discrimination but also their activity has, in turn, a bidirectional function on memory generalization specifically during retrieval of object-context experiences.

**Comment 2.6:** On several occasions, the authors relate their work to episodic memory but the work does not reflect episode of information at all. Statements such as in the abstract last sentence:
'They also provide new insights into molecular mechanisms underlying the communication between hippocampal subregions', is overestimated because this study does not provide any molecular mechanisms explain inter-hippocampal communication. There are several similar locations within the text, which require a thorough revision of the text.  I final criticism is the embedding of the single unit recording data. They seem to be much unrelated to the rest of the manuscript.

Response to comment 2.6: We agree with the Reviewer that it was incorrect to refer to “communication between hippocampal subregions'', since we address the contribution of CA3 and DG independently and do not provide results regarding the communication between DG and CA3. For this reason, we have replaced this sentence in the revised version of the manuscript to “They also provide new insights into the molecular mechanisms underlying the contributions of hippocampal subregions to generate this balance”. We understand why the reviewer may find that the reference to “episodic memory” is an overstatement. It is certain that we can only speak of object in context memories having no measure of the temporal aspect of episodic memory. However, we do believe we have shown in the manuscript that these events constitute an episode of object in context memory, where spatial information is bound to object information. The fact that the contextual experience alone is able to reactivate the object memory even in the absence of any object, making it susceptible to Emetine (Fig. 2E, 4D) speaks clearly on this behalf. In accordance with this, we have changed references to “episodic memories'' to “object in context” memories. Although the existence of episodic types of memories in rodents has been debated [14-17], it is clear that spontaneous object recognition tasks model some features of it. For example, object in context memories include the “what” and “which context” aspects of episodic memory, but not the “when” [18-21]. Thus, we believe this study is actually relevant to the understanding of episodic memory mechanisms.

Additionally, to add coherence to the embedding of the single unit recording data into manuscript, we have added the following paragraph explaining what is known in the field about the link between spatial memory and place fields, and which is the gap we are trying to fill with our experiments:

*“While several studies have shown that different experiences can be represented in the hippocampus [41, 42], a direct link between the individual’s internal context representation and memory retrieval is still missing. Taking advantage of our new behavioral paradigm, which allows us to measure how well animals remember a given context, we aimed at addressing an important, yet unresolved question: how is place cell coding (in particular remapping) related to spatial memory? To assess the relationship between contextual representation and object in context memory (...).”* (see page 4, line 191)

**Comment 2.7:** The authors argue that exposure of the object within an enriched (AC) context requires protein synthesis in the perirhinal cortex (PrC) because infusion of the PrC with Emetine reduces object discrimination if the familiarized object is combined in the AC context with a novel object. Thus, this is an object recognition task discrimination is based on the recognition of the novel previously unexperienced object. The less cues are available in the environment the more seems the animal to focus on the object. In a cue-lacking environment the animals has nothing else to do than to explore the only object in the arena present. Thus, it is not necessarily the object-context relationship, which drives the stronger observation of the object but the pure lack of any other visual or tactile inputs.

**Response 2.7:** We thank the reviewer for this comment. We apologize again for not being clear with the protocol of our experiments on Fig. 2. The manipulation of the number of cues was only done during the TS1, and not when the objects were present (TS2). In the same manner, the Emetine infusion was done the day when the object was not presented. The rationale behind our conclusions stands from the idea that, in the absence of any object present, it can only be the mnemonic association between the object and the context that can make the object memory susceptible to Emetine by the mere presentation of an AC context.

No tradeoff between the time the animal spends in the context and the time spent exploring the object could explain our results since, as explained on response to comment 2.2, in both the AC and NC groups there is no object during the TS1 (the only moment where there is a manipulation of contextual cues).

**Comment 2.8:** The triangles in Figure 2 are confusing, because triangular environments are shown with the label AC implying that animals are exposed to enriched environments. Thus, external cues should be present in this part of the figure.

**Response 2.8:** We thank the Reviewer for pointing this out. It is important to mention that animals were not exposed to AC or NC conditions in the triangular environment, but only in the circular environment. The triangular context has no contextual cues and no relationship with the circular arena where the objects were originally experienced. The experiment was designed in this way to evaluate the memory of the objects independently of its context. Therefore, the lack of external cues in this part of the figure is intentional. Indeed, **the label AC at the end was intended to represent the label assigned to the group that went through a retrieval session on AC conditions during TS1**, and does not refer to their condition in TS2 (that is exactly the same for both groups). We realize now that the protocol schema we used on Fig.2A could lead to this misinterpretation since the word AC is at the end of the schema, and we apologize for this mistake. We have corrected the schema in the final manuscript by moving the position of this label on Fig.4D to avoid any misunderstanding. Moreover, we added a more thorough explanation of the protocol in the Results Section (Page 4 and 6), as following:

“*To address this question, animals trained on day 1 in the task were exposed to a retrieval session on day 2 in the same context but in the absence of an object in either AC or NC conditions (TS1). Immediately after context exposure, they were infused into the Prh with the protein synthesis inhibitor Emetine (Eme) or with Vehicle (Veh). Object memory was evaluated 24 hours later (day 3) on a test session (TS2), by placing the original object next to a novel one on a novel triangular context lacking any contextual cues so that object memory could be estimated independently of any contextual contribution (Fig. 2A, see Methods section).”* (see page 4, line 159)

*“(...) For this, after training on a context-object association as done before (day 1), animals infused with either AP5 or Veh in CA3 were subjected to an AC contextual re-exposure in the absence of any object, and Emetine or Veh were infused in the Prh immediately after (TS1). On day 3, the original object memory of day 1 was tested on the triangular context against a novel object (TS2).”* (see page 6, line 300)

**Comment 2.9:** It remain unclear why Emetine was injected in the perirhinal cortex because recent studies showed that context memory is encoded and stored within the dentate gyrus for several days up to weeks and infant memories can be recalled upon reactivation of principal cell assemblies in the adult rodent (Kim et al., Nature Comm 2020; Guskjolen et al., Curr Biol, 2018). Thus, the dentate gyrus seem to keep contextual information for long periods of time. What would have happened on the behavioral level if Emetine was injected in the DG or in a different brain area not involved in the binding of objects and context?

**Response 2.9:** We thank the Reviewer for the opportunity to address this point. We wanted to show that animals were forming object in context memories in our paradigm. For this, we tested whether the contexts only could lead to the retrieval of a memory that incorporates both the what (ie. object) and the where (ie. context). To evaluate the effect over object memories independently of the context, animals were tested for the memory of the object in a triangular arena in which the objects were not previously experienced. Moreover, the use of Emetine Prh infusions to target object memories that were associated with a particular context has been published in the past by our team, and we were able to show that contextual information can drive object memory reconsolidation in Prh [10].

In conclusion, the Emetine injection in the Prh was intended to interfere with the object memory and not with the spatial memory. This is why we interfered with a region that was shown to be important for object memory consolidation and reconsolidation [1, 10, 22]. We intentionally avoided interfering with regions that are linked to spatial memory and that classically have a more questioned role for object memory. Although we could speculate that Emetine in the DG could have an effect over the object memory per se (although smaller when compared to the Prh injection), we believe that it was much more relevant to inject Emetine in the Prh targeting the object memory and not the spatial memory.

**Comment 2.10:** One of the main conclusions of Figure 2 is that the context is able to guide reactivation of 'relevant' object memories, but what is here a 'relevant' object memory? Indeed the data shown in Figure 2 indicate that the second recall of the enriched context is important to make an association between the object and the context and allows the identification of the novel object. It has been previously shown in object recognition tasks that 24 h after initial environmental exposure memory is stored and can be recalled in object recognition tasks. It is also very clear from the literature that distal cues within the environment are important to identify a context and help the animal to memorize a given context. Thus, exposure of rodents to a NC condition with lacking cues makes a 'remembering' or consolidation of a memory quite difficult. Therefore some of the here presented results are not surprising.

**Response 2.10:** We have likely explained this issue in response to previous comments, but we can reinforce our clarification. In Figures 2C-E we show that, despite having no additional visual cues, the NC condition still provides contextual information and can be used to guide retrieval of associated objects. Critically, it can only act as a retrieval cue for information that is associated with that NC context (ie. the object experienced in that context, represented by the star) and not for information associated with the same context but with distal cues as in AC (represented by a circle). What our results show is that exposure to either NC and AC can labilize the object in context memory (in the absence of any object). However, there is a difference in terms of ‘which’ object memory is affected. For the NC context exposure, only the object experienced in NC on day 1 was affected by Emetine (and not the object experienced in AC the same day), while the opposite is the case for the AC. We believe this is strong evidence to support that contexts, independently of the number of distal visual cues, are guiding the retrieval of each object in a specific manner.

**Comment 2.11:** Another finding of the study is that spatial correlations were higher in the enriched AC context than the 'no cue' (NC) context. It has been previously shown by Kentros and Kandel that the higher the number of external cues within an environment the more place cells are formed and the higher is the spatial tuning of the principal cells. The enrichment of the scenery helps the animal to orient and to identify a given context. It's therefore not surprising (and not novel) that the here observed relationship between spatial tuning and contextual setting are related. It is also not surprising that the spatial correlations decline in sessions of higher object exploration because most of the activity will be surrounding the explored object and mice / rats run less around the arena to explore the environment.

**Response 2.11:** We thank the Reviewer for this contribution. We compared the percentage of place cells out of the total number of neurons recorded in each condition and found no significant differences. We also compared the spatial information index and the size of the place field in each condition and found no differences between groups. We have included these analyses in Fig. S.6A-C. These results suggest that, in our task, the reported effect in spatial correlation and firing rate change is not a consequence of place cells properties due to differences in the number of visual cues in the context. In addition, we have included in Fig. S.4 an analysis of the total distance traveled, the mean instantaneous velocity and a comparison of the time spent in each spatial bin (occupancy) between phases in each condition. We also found no differences. Rats explore the context uniformly among conditions, so the differences found in place cell activity could not be a consequence of differential context exploration. We do not agree with the reviewer's interpretation that our results are similar to what Kentros and Kandel found and thus, they are trivial or not novel. We believe our results do not match the findings of *Kentros et al.*, but we can only speculate why this happened. Importantly, that particular study recorded place cells from CA1, not CA3 like in the present manuscript, so those results are not easily comparable with ours. Having said this, we did not observe more place cells in the AC compared with the NC context, so just the absence of the removable visual cues in the NC context cannot explain the lower correlation between place cell maps between these conditions. We argue that it is the internal contextual representation that differs in animals that discriminate between these conditions and not the lack of visual “enrichment”. One detail is that the AC, PC and NC contexts are not that different to each other. The 6 distal visual cues are likely not the only cues animals use to orient themselves in the arena. We think this is why we were able to disentangle the internal representation from the external condition in the electrophysiology experiments. Despite the difference in the distal removable visual cues, there is conserved ambiguity between the contexts that is reflected in place cell activity and also in behavior. We thank the reviewer for the opportunity to address what we believe is an interesting point, and for this reason we have added a paragraph on this regard on the Discussion, as following:

*“Our results contrast with those of Kentros et al. (2004), who described an increase in the number and tuning of place cells when the number of environmental cues is higher.* *Importantly, that particular study recorded place cells from CA1, not CA3 like in the present manuscript, so those results are not easily comparable with ours. Having said this, in our task the 6 distal visual cues are likely not the only cues animals use to orient themselves in the arena, this could make the AC and NC context more similar in terms of their contextual representation. This might be the reason why we can disentangle the internal representation from the external condition in the electrophysiology experiments. Despite the difference in the distal removable visual cues, there is conserved ambiguity between the contexts that is reflected in place cell activity and also in behavior.”* (see page 9, line 441)

**Comment 2.12:** Another proposed novel observation also relates to Figure 3: place cells encode more than a field in the environment but also the context. This observation is absolutely known for a substantial time and went in the literature as the 'spatial' and 'context code'.

**Response 2.12:** Again, we respectfully disagree with the reviewer regarding the novelty of our findings. Although several studies suggest the existence of a ‘spatial’ and a ‘contextual’ code, most of them lack a clear connection between place cell activity and animal behavior. In this study, we provide evidence of the contextual coding of the hippocampus showing a direct link between place cells remapping and episodic-like memory expression. Our behavioral task combined with the electrophysiological recordings, allows us to have a read-out of memory expression while recording place cell activity, something that has not been extensively shown to date.

**Comment 2.13:** Figure 4 indicates that NMDA receptor activation is needed for the storage and recall of object memories if the environment is equipped with a low number of external cues (PC condition), but how is the situation in the 'no cue' environment? If the authors are right with their assumption than we would expect that under NC conditions, the recruitment of NMDA receptors should be more important than under PC conditions and expressed in high object exploration times during AP5 application (Figure 4C).

**Response 2.13:** Although we do believe that NMDAR activation in CA3 is important for memory retrieval under partial cue conditions, we also think NMDAR activation by itself is not enough for memory retrieval. We propose that our pharmacological manipulation modulates memories only if they are already susceptible to retrieval (ie. if external input can potentially act as a retrieval cue). This was the rationale for using a partial cue condition and not a context that lacks any distal cue. The lack of distal cues in NC could prevent this context from acting in most animals as a cue for retrieving the original object in context memory. If the NC is able to act as an even more degraded cue for retrieval, AP5 in CA3 for the NC condition should prevent memory generalization and lead to higher exploration levels. However, a practical problem with this experiment is that NC animals already show on average high levels of object exploration, making it less likely for our manipulation to be able to evidence this contribution due to a possible roof effect on exploration time. Although we might see some individual changes for the small percentage of animals that do show retrieval even in NC, on a population level there would probably be no significant effect.

**Comment 2.14:** NMDA receptor blockade showed (Figure 5) that the ability of the DG to discriminate between two similar but distinct contexts is markedly diminished. It is concluded that NMDA receptors play a key role in pattern separation (orthogonalization) processes. Since the publication from McHugh et al (Nature 2007), this observation is well perceived and broadly accepted. In this respect, the reviewer therefore cannot identify any novel additional information within this study.
The NMDA receptor agonist increased exploration time. Please provide information on the running velocity and path length. Could it be that via granule cells of the DG are more recruited by this agonist and thus provide a more general increase in neuronal excitation to downstream hippocampal areas, which cause an enhanced mobility of the animal?

**Response 2.14:** This is certainly an important point. Even though NMDARs on the DG were previously linked to pattern separation, this process has been studied independently of memory retrieval. Therefore, how this mechanism could contribute to memory generalization has never been addressed. The novelty in our study is that we showed that this mechanism not only contributes to independent cognitive functions such as pattern separation, but they instead regulate the balance between memory discrimination and generalization or between encoding and retrieval. With respect to the possibility that NMDAR agonists increase neuronal excitation and lead to a higher mobility of the animal, this seems unlikely. We have shown that infusion of D-Cyc in the DG or CA3 region before exposure to an object in the AC context did not alter exploration compared with Vehicle-infused groups (see page 7, line 367). These results already suggest that the effect we found is related to memory retrieval. We appreciate the suggestion of the Reviewer of complementing this information by adding information of the distance traveled by the animal during the task on the DG D-Cyc experiment. We have tracked the animals and found that there was no difference in the %distance traveled or in the linear velocity per second between D-Cyc and Veh injected animals during the test session, neither when D-Cyc was injected in the DG nor in CA3. We have added this new information on Fig S3. in the new version of the manuscript. We also commented this result in the results section as following:

*“It is important to note that animals infused with D-Cyc showed no increase compared to Veh injected animals in the distance travelled or linear velocity during test session compared to Veh injected animals. This outcome rules out an effect of D-Cyc over general mobility levels (Fig. S4)."* (see page 7, line 346)

**Comment 2.15:** Minor: Use similar y-axis scales such as in Figure 1D and G.

**Response 2.15:** We thank the Reviewer for pointing this out. We agree that the scales should be similar and we have revised the manuscript for scale differences which were corrected in the new version.

**Comment 2.16:** Minor: Explain better the triangle environment in Figure 2 and 4.

**Response 2.16:** As stated on Response 2.2, we have added a paragraph explaining in more detail the triangular environment and in what conditions object memory was tested in TS2.

**Comment 2.17:** Minor: If statistical comparisons were not significant, than please indicate this with an 'NS'.

**Response 2.17:** We appreciate the Reviewer comment. Non-significant statistical comparisons have been added to the graphs on the final version of the manuscript.

**Comment 2.18:** Minor: The bar graphs could be made a bit nice (similar thickness of lines, boarder of the bars in Figure 1 show different color than the bar filling).

**Response 2.18:** We thank the Reviewer’s comment. We have changed the borders of the graphs to the same color as the bar and homogenize the line thickness across graphs.

**RESPONSE TO REVIEWER 3**

**Comment 3.1:** Miranda and colleagues investigate the role of CA3 place cell remapping and NMDAR activity in CA3 and DG in an incidental object-context recognition task.  The authors find that memory for object-context associations can be predicted by CA3 remapping and is differentially influenced by NMDAR activity in CA3 and DG.

Although remapping, and its theoretical relation to key computational processes implicated in memory (pattern separation and completion), has been studied extensively over the past decades not many studies have attempted to relate remapping to the behavioural expression of pattern completion and separation.  Further, their pharmacological manipulations provide added evidence to the role of NMDA receptors in hippocampal memory and the complementary role of CA3 and DG.

On the whole, I found the manuscript easy to follow and I think the authors' conclusions are largely supported by their results.  However, there are parts of the main text that I feel lack clarity, and I think the CA3 correlation analysis could be expanded considerably to better understand CA3 population dynamics in pattern completion. Finally, some key comparisons seem to be missing from their pharmacological experiments. 

I detail my specific comments below.  All of the suggestions are easily addressable with the current dataset and I think with the additions and revisions the impact of the manuscript would be significantly enhanced.

**Response 3.1:** We are pleased that the Reviewer finds our work novel and easy to follow, and our conclusions well supported by the results.

**Comment 3.2:** The authors state they find no difference in object exploration for different cue conditions during training (Fig1C,F).  But why were there different cue conditions used during training? Figure 1 shows that during training the environment has 6 distal cues and then during testing the environment either had the same number of cues (AC), a partial set (AP) or no distal cues (NC).  Did they include extra training groups to make sure the number of cues in of itself could explain exploration times during testing?  If so, the test group they analyse in all of their main analysis is the group that got the AC condition in training, no?  Some clarity on the task procedures would be helpful, and if the authors indeed used control training groups for PC and NC, this should be explicitly stated.

**Response 3.2:** We welcome the Reviewer’s insightful query and we can certainly clarify this issue. There were no differences in the cue conditions during training. All the cue manipulations were performed during testing with no exception. All groups were trained in AC condition. However, we did control for a priori differences between groups in terms of exploration during training (even if they all were subjected to the exact same training conditions and will only be treated differently afterwards). This is why we stated that we found no difference in object exploration for different cue conditions during training. We understand that this phrase could lead to a misunderstanding. For this reason, we have pointed out this part of the procedure more clearly in the new version of the manuscript. Specifically, the following passage has been introduced in the “Results'' section:

*“We found no difference in object exploration time between groups during training (Fig. 1C, 1F). Since all groups were trained on AC conditions, this evidences all groups were a priori equal in terms of object exploration levels.”* (see page 3, line 141)

We have also added a paragraph in the Methods section after explaining each test condition, as following:

*“For all groups, the training phase was done on AC conditions, in which all 6 distal cues were present.”* (see page 12, line 634)

**Comment 3.3:** The authors show that exploration time can be fitted with two gaussian distributions, one that captures enhanced exploration (when a change is detected, signaling encoding) and another that captures reduced exploration (signaling the animal treats the environment as the same as during training, hence retrieval). It would be useful to see the data distribution used to fit the gaussian components with the gaussian components indicated as well.
Further, it would be insightful to do a similar analysis of the spatial correlation/firing rate change data. Namely, combining correlation/rate change data from all session types, is it apparent that the data effectively contains two distributions, one with a low spatial correlation/high rate change when the animal explores the object during test (=encoding) and another with a high spatial correlation/low rate change when the animal explores the object less during test (=retrieval)?  Showing the distributions and the contributions of each component would give insight into the homogeneity of CA3 cell responses and the population dynamics that underlie the switch between a memory encoding vs retrieval state (pattern separation vs completion).  For example, for test sessions with high exploration do you only see the low spatial correlation component? Alternatively, do you see both components but the low correlation component dominates? One may hypothesise the opposite to be true for test sessions with low object exploration.

**Response 3.3:** We thank the reviewer for this comment. We included a graph showing the distribution of behavioral data with the two fitted Gaussians in Supplementary Figure 7A, but it wasn't correctly referenced in the main text. We apologized for not being clear with the reference, we have now correctly referenced this figure in the page 5 line 236**.**

Following the reviewer suggestion, we performed a similar analysis with the spatial correlation and the firing rate change. Using both variables, we were able to classify place cells into two populations. These two populations are clearly different, one cluster (cluster A in the text) has high spatial correlation and low firing rate change, with a higher proportion of place cells from sessions where animals have a low exploration of the object (memory retrieval). The other cluster (cluster B) is the opposite, place cells belonging to this cluster have lower values of spatial correlation and higher firing rate change. Interestingly, in sessions in which the animals retrieved the original memory there was a higher proportion of cluster A than cluster B neurons, and vice versa, when animals perceived the context as different, there was a higher proportion of cluster B cells. This new result is now reported in the Figure 3H-J and in the text:

*“To better understand how the neuronal population might represent this dichotomic behavioral output, we examine whether our neuronal population can be classified according to its spatial correlation and firing rate change values. To do this, we computed t-SNE (t-distributed stochastic neighbor embedding) over the two variables and performed a k-mean clustering, finding two clear clusters (evaluated by the Silhouette method). Cluster A has a higher mean spatial correlation than Cluster B (Fig.3I, n=165-167, LMM, p=1.9186e-63) and a lower firing rate change (Fig. 3I, n=165-167, LMM, p= 1.0141e-14). Looking at the proportion of each cluster according to the internal representation, we found that Cluster B is more represented in the non-retrieval session (Fig. 3J, 80%, 4 out of 5) and Cluster A is more represented in the retrieval sessions (Fig. 3J, 75%, 12 out of 16, X2 (1, N = 21) = 4.887, p = .0271). In addition, most cells from the session where animals had a different internal representation (79%) are in Cluster B. This suggests that Cluster B grouped neurons represent a change in the animal’s internal representation. In summary, when animals are unable to recall the initial memory, there is a greater representation of cells from Cluster B. These cells exhibit a low spatial correlation and a high firing rate change.”*(page 5, line 251-264).

**Comment 3.4:** Figure 3 shows example ratemaps that show different degrees of CA3 remapping. I was struck by the low spatial correlation for the PC condition. This seems somewhat at odds with the summary statistics figures (fig3b) that shows PC condition has similar correlations to the AC condition. Is this example somewhat of an outlier? In this vein, it would be useful to see some more examples of CA3 ratemaps so the reader can get an impression of the nature and variability in remapping.  Seeing this low correlation for the PC condition also underscores the utility of analyzing the remapping distributions as suggested in the previous point.

        Relatedly, it would be useful to also normalize the TR and TS ratemaps so that the peak is the same. This will bring out more clearly rate remapping.

**Response 3.4:** We thank the reviewer for pointing this out; indeed, the example shown for the PC condition is not representative of the majority of place cell responses in this condition. We have added more examples of CA3 rate maps for all conditions in Figure 3 to clearly show the place cell responses. In addition, we included in Figure supplementary 5A the rate maps of the same place cells shown in the figure 3 but normalized to the rate map with maximum firing rate.

**Comment 3.5:** The use of abbreviations, signaling different cue conditions, testing vs training, etc, makes the text a little hard to follow. Can the authors inter-mingle the use of their abbreviations with sentences that actually just describe in normal terms what the results show.  e.g. the following sentence is quite hard to parse (and I think contains a typo on in the abbreviation): "In accordance with this, the proportion of animals that show high exploration consistent with non-retrieval in test (in red) varies from 0-18% in TC and CP Veh injected sessions to 50% in the CP AP5 injected test sessions"

**Response 3.5:** We welcome the Reviewer’s suggestion. We have thoroughly revised the manuscript for sentences that are difficult to follow. We have rephrased the abbreviations in long sentences. Examples of sentences that have now been rewritten are shown as follows:

*“On the other hand, Emetine infusion in the Prh significantly decreased the discrimination ratio in the “all cue” condition when compared to Vehicle, while no effect was seen in the “no cue” condition”.* (see page 4, line 167)

*“Nevertheless, animals infused with Emetine after “no cue” exposure only showed a reduction of the discrimination ratio for the object originally associated with that condition. On the other side, animals infused with Emetine after “all cue” condition had selective reduction for the discrimination of the object associated with AC during the training session.”* (see page 4, line 179)

*“In accordance with this, the proportion of animals that show high exploration consistent with non-retrieval in test (in red) varies from 0-18% in “all cue” TC and “partial cue”CP Vehicle injected sessions to 50% in the “partial cue”CP AP5 injected test sessions.”* (see page 6, line 294)

**Comment 3.6:** It is interesting that the animals preference for pattern completion or separation can be manipulated by altering the activity of NMDAR in CA3 and DG.  Their main experimental condition is the partial cue condition, however could they not induce pattern separation/completion in the NC condition with their pharmacological manipulation.  e.g. if the DG NMDARs are inactivated, will the animals then also generalize the NC context?

**Response 3.6:** This is a very interesting comment. We have never attempted to induce pattern completion in the NC condition. Although we believe that inactivation of NMDARs in the DG can modulate memory retrieval, we also understand that this inactivation by itself is not enough. There is also a requirement for external contextual inputs with the potentiality to act as a cue for retrieval on the DG-CA3 system in order to sustain memory generalization, even in the presence of this pharmacological modulation. The NC condition has the risk of lacking these minimal distal cues to activate retrieval. This could potentially prevent this context from acting as a cue for retrieval even under pharmacological modulation. For this reason, we decided to perform those experiments on an intermediate PC2 condition instead of using the NC condition. It is notwithstanding possible that even under a higher reduction in the number of cues DG NMDAR inactivation will still lead to an increase in the level of generalization. In fact, given our results on place cell remapping, depending on the internal state, they could be susceptible to NMDAR blockade in the DG in a test session using the NC condition. It is likely that the number of subjects required to see this effect is quite high. However, we can only speculate in this regard. We have added a paragraph in the “Discussion” section to address this, as follows:

*“Although inactivation of NMDARs in the DG can modulate memory retrieval, it is likely that inactivation of NMDARs in the DG is not sufficient to support memory retrieval by itself. Rather, a requirement for external contextual inputs with the potentiality to act as a cue for retrieval on the DG-CA3 system is also needed in order to sustain memory generalization (even under this modulation). In this regard, it is possible that an absence of contextual cues, as could be the case on the NC condition, could prevent a context from acting as a cue for retrieval. Only further experiments will be able to tell us whether even under a higher reduction in the number of cues, DG NMDAR inactivation will still lead to an increase in the level of generalization.”* (see page 10, line 520)

**Comment 3.7:** Minor: The use of the word previous in multiple places in the text should be replaced with prior (e.g. "Animals were infused inCA3 with the L-type calcium channel inhibitor Nimodipine (Nim) *prior* to the D-Cyc infusion and then tested in the PC2 context"

**Response 3.7:** We thank the reviewer for his/her suggestion. We have now rephrased our excessive use of the word ‘previous’ for the word ‘prior’.

**RESPONSE TO EDITOR**

Editor: “One specific suggestion that came up is to control for the specific pattern of sampling between the TR and TS sessions by down-sampling the data to match the minimum number of samples at each visited location when comparing rate maps, as explained in (Keinath et al., 2018, eLife), to ensure that you’re not simply reading out the behavioral biases in location.”

We appreciate the opportunity to address the editor's concerns. We have controlled for animal behavior across conditions and found no differences in the total distance traveled and the mean instantaneous velocity between conditions. We also computed the Pearson correlation between the occupancy maps (maps of the amount of time the animals spent in each bin) of the training and test phases and found no differences between conditions. We included these analyses in the Supplementary Figure 4A-C. These results suggest that the overall contextual exploratory behavior remains consistent across conditions. In addition, when computing the spatial correlation and the firing rate change, only bins visited more than 4 times and for more than 15 ms in each phase are included, so if a bin has low exploration in either of the two phases that bin is not included in the analysis. Nevertheless, we include some examples, in the Supplementary Figure 5B, using the subsampling method described in *Keinath et al., 2018*, which show quite similar place maps and remapping parameters compared to the method used for the full analysis.

**References**

1.	Winters BD, Bussey TJ. Transient inactivation of perirhinal cortex disrupts encoding, retrieval, and consolidation of object recognition memory. J Neurosci. 2005;25(1):52-61. doi: 10.1523/JNEUROSCI.3827-04.2005. PubMed PMID: 15634766.

2.	Balderas I, Rodriguez-Ortiz CJ, Salgado-Tonda P, Chavez-Hurtado J, McGaugh JL, Bermudez-Rattoni F. The consolidation of object and context recognition memory involve different regions of the temporal lobe. Learn Mem. 2008;15(9):618-24. doi: 10.1101/lm.1028008. PubMed PMID: 18723431; PubMed Central PMCID: PMCPMC2632790.

3.	Miranda M, Morici JF, Gallo F, Piromalli Girado D, Weisstaub NV, Bekinschtein P. Molecular mechanisms within the dentate gyrus and the perirhinal cortex interact during discrimination of similar nonspatial memories. Hippocampus. 2021;31(2):140-55. doi: 10.1002/hipo.23269. PubMed PMID: 33064924.

4.	Jessberger S, Clark RE, Broadbent NJ, Clemenson GD, Jr., Consiglio A, Lie DC, et al. Dentate gyrus-specific knockdown of adult neurogenesis impairs spatial and object recognition memory in adult rats. Learn Mem. 2009;16(2):147-54. doi: 10.1101/lm.1172609. PubMed PMID: 19181621; PubMed Central PMCID: PMCPMC2661246.

5.	McHugh TJ, Jones MW, Quinn JJ, Balthasar N, Coppari R, Elmquist JK, et al. Dentate gyrus NMDA receptors mediate rapid pattern separation in the hippocampal network. Science. 2007;317(5834):94-9. doi: 10.1126/science.1140263. PubMed PMID: 17556551.

6.	Neunuebel JP, Knierim JJ. CA3 retrieves coherent representations from degraded input: direct evidence for CA3 pattern completion and dentate gyrus pattern separation. Neuron. 2014;81(2):416-27. doi: 10.1016/j.neuron.2013.11.017. PubMed PMID: 24462102; PubMed Central PMCID: PMCPMC3904133.

7.	Nakashiba T, Cushman JD, Pelkey KA, Renaudineau S, Buhl DL, McHugh TJ, et al. Young dentate granule cells mediate pattern separation, whereas old granule cells facilitate pattern completion. Cell. 2012;149(1):188-201. doi: 10.1016/j.cell.2012.01.046. PubMed PMID: 22365813; PubMed Central PMCID: PMCPMC3319279.

8.	Sahay A, Scobie KN, Hill AS, O'Carroll CM, Kheirbek MA, Burghardt NS, et al. Increasing adult hippocampal neurogenesis is sufficient to improve pattern separation. Nature. 2011;472(7344):466-70. doi: 10.1038/nature09817. PubMed PMID: 21460835; PubMed Central PMCID: PMCPMC3084370.

9.	Kubik S, Miyashita T, Guzowski JF. Using immediate-early genes to map hippocampal subregional functions. Learn Mem. 2007;14(11):758-70. doi: 10.1101/lm.698107. PubMed PMID: 18007019.

10.	Morici JF, Miranda M, Gallo FT, Zanoni B, Bekinschtein P, Weisstaub NV. 5-HT2a receptor in mPFC influences context-guided reconsolidation of object memory in perirhinal cortex. Elife. 2018;7. doi: 10.7554/eLife.33746. PubMed PMID: 29717980; PubMed Central PMCID: PMCPMC5931799.

11.	Keinath AT, Nieto-Posadas A, Robinson JC, Brandon MP. DG-CA3 circuitry mediates hippocampal representations of latent information. Nat Commun. 2020;11(1):3026. doi: 10.1038/s41467-020-16825-1. PubMed PMID: 32541860; PubMed Central PMCID: PMCPMC7296021.

12.	Sun C, Yang W, Martin J, Tonegawa S. Hippocampal neurons represent events as transferable units of experience. Nat Neurosci. 2020;23(5):651-63. doi: 10.1038/s41593-020-0614-x. PubMed PMID: 32251386.

13.	Nakazawa K, Quirk MC, Chitwood RA, Watanabe M, Yeckel MF, Sun LD, et al. Requirement for hippocampal CA3 NMDA receptors in associative memory recall. Science. 2002;297(5579):211-8. doi: 10.1126/science.1071795. PubMed PMID: 12040087; PubMed Central PMCID: PMCPMC2877140.

14.	Tulving E. Episodic memory: from mind to brain. Annu Rev Psychol. 2002;53:1-25. doi: 10.1146/annurev.psych.53.100901.135114. PubMed PMID: 11752477.

15.	Roberts WA, Feeney MC, Macpherson K, Petter M, McMillan N, Musolino E. Episodic-like memory in rats: is it based on when or how long ago? Science. 2008;320(5872):113-5. doi: 10.1126/science.1152709. PubMed PMID: 18388296.

16.	Suddendorf T, Corballis MC. Mental time travel and the evolution of the human mind. Genet Soc Gen Psychol Monogr. 1997;123(2):133-67. PubMed PMID: 9204544.

17.	Huston JP, Chao OY. Probing the nature of episodic memory in rodents. Neurosci Biobehav Rev. 2023;144:104930. doi: 10.1016/j.neubiorev.2022.104930. PubMed PMID: 36544301.

18.	Chao OY, Huston JP, Li JS, Wang AL, de Souza Silva MA. The medial prefrontal cortex-lateral entorhinal cortex circuit is essential for episodic-like memory and associative object-recognition. Hippocampus. 2016;26(5):633-45. doi: 10.1002/hipo.22547. PubMed PMID: 26501829.

19.	Dere E, Huston JP, De Souza Silva MA. Integrated memory for objects, places, and temporal order: evidence for episodic-like memory in mice. Neurobiol Learn Mem. 2005;84(3):214-21. doi: 10.1016/j.nlm.2005.07.002. PubMed PMID: 16102980.

20.	Langston RF, Wood ER. Associative recognition and the hippocampus: differential effects of hippocampal lesions on object-place, object-context and object-place-context memory. Hippocampus. 2010;20(10):1139-53. doi: 10.1002/hipo.20714. PubMed PMID: 19847786.

21.	Sep MSC, Vellinga M, Sarabdjitsingh RA, Joels M. The rodent object-in-context task: A systematic review and meta-analysis of important variables. PLoS One. 2021;16(7):e0249102. doi: 10.1371/journal.pone.0249102. PubMed PMID: 34270575; PubMed Central PMCID: PMCPMC8284613.

22.	Balderas I, Morin JP, Rodriguez-Ortiz CJ, Bermudez-Rattoni F. Muscarinic receptors activity in the perirhinal cortex and hippocampus has differential involvement in the formation of recognition memory. Neurobiol Learn Mem. 2012;97(4):418-24. doi: 10.1016/j.nlm.2012.03.006. PubMed PMID: 22452926.

23.	Kentros CG, Agnihotri NT, Streater S, Hawkins RD, Kandel ER. Increased attention to spatial context increases both place field stability and spatial memory. Neuron. 2004;42(2):283-95. doi: 10.1016/s0896-6273(04)00192-8. PubMed PMID: 15091343.

24.	Keinath AT, Epstein RA, Balasubramanian V. Environmental deformations dynamically shift the grid cell spatial metric. Elife. 2018;7. doi: 10.7554/eLife.38169. PubMed PMID: 30346272; PubMed Central PMCID: PMCPMC6203432.
